# Early Predictors of the Childhood Dysregulation Profile: A Systematic Review

**DOI:** 10.1007/s10567-026-00557-7

**Published:** 2026-02-06

**Authors:** Elzbieta Vitkauskaite, Ayten Bilgin

**Affiliations:** 1 Department of Health and Social Care, University of Essex, Colchester, UK; 2 Department of Psychology, University of Essex, Colchester, CO4 3SQ UK; 3 Department of Psychology, University of Warwick, Coventry, UK

**Keywords:** Childhood dysregulation profile, Childhood psychopathology, Early predictors, Systematic review

## Abstract

Childhood dysregulation profile (DP) involves difficulties in regulating emotions, behaviour and cognitions, and is associated with adverse long-term outcomes, yet its early life predictors are less well understood. This systematic review examined peer-reviewed studies published in English up to December 2024, identifying 12 eligible articles. Findings revealed that family-related factors such as parental mental health symptoms, lower education, prenatal substance use, and higher social adversity were associated with increased childhood DP symptoms, whereas evidence on parenting behaviours and home environment was inconclusive. Among child-related predictors, boys, children with difficult temperaments, and those with early regulatory problems (i.e., excessive crying, sleeping or feeding problems) were more likely to show childhood DP symptoms. On the other hand, the impacts of low birth weight, and gestational age were inconclusive. Research on the influence of language, cognitive skills and early social development remains limited. Further prospective longitudinal studies are needed to strengthen the evidence base.

## Introduction

Children who struggle to regulate their emotions, behaviour, and cognitions may present with a dysregulation profile (DP), a broad construct of childhood psychopathology rather than a clinical diagnosis (Althoff, et al., 2010). It is characterised by combined elevated symptoms of emotional problems (depression, anxiety), hyperactivity/ inattention, and conduct problems (e.g., aggression) (Althoff et al., 2010; Ayer et al., 2009). This differs from emotion dysregulation, which refers more narrowly to difficulties in managing, modulating, and responding to emotional experiences (Thompson, 2019). Emotion dysregulation could occur on its own, whereas DP reflects a broader and more severe pattern that includes emotional symptoms alongside hyperactivity/inattention, and conduct problems. Its global prevalence ranges from 2 to 18% (Rescorla et al., 2021). Consistent evidence suggests that childhood DP is a risk factor for later psychopathology (Caro-Cañizares et al., 2019; Deutz et al., 2016; Masi et al., 2015). However, early contributing factors to the development of childhood DP symptoms remain less understood. Identifying these factors is crucial for early intervention, prevention, and more effective treatment, potentially reducing long-term psychological consequences.

### Childhood Dysregulation Profile: Definition and Assessment

Childhood DP was initially conceptualized as an indicator of the juvenile/paediatric bipolar disorder (Biederman et al., 1995). However, later research found that DP is not uniquely associated with symptoms of bipolar disorders in children but instead reflects a broader vulnerability to various forms of psychopathology (Ayer et al., 2009). As interest in dysregulation-related disorders has grown among researchers and clinicians, these disorders (though not childhood DP specifically) have been recognized in the most recent versions of major diagnostic systems. The Diagnostic and Statistical Manual of Mental Disorders (DSM-5; American Psychiatric Association, 2013) includes childhood DP as Disruptive Mood Dysregulation Disorder (F34.8) within the depressive disorders category, describing children with severe irritability and anger. Similarly, the International Classification of Diseases (ICD-11; World Health Organization, 2018) includes childhood DP as Oppositional Defiant Disorder (ODD) with chronic irritability-anger (6C90.0) under disruptive behaviour or dissocial disorders as a subtype of ODD characterized by prevailing, persistent angry or irritable mood.

Previous research has commonly assessed childhood DP using a combination of three subscales from the Child Behaviour Checklist (CBCL): anxious/depressed, aggressive behaviour and attention problems. The Strengths and Difficulties Questionnaire (SDQ; Goodman, 1997) has also been identified as a valid psychometric tool for measuring childhood DP (Holtmann et al., 2011a). The CBCL and SDQ are similar tools as their subscales are highly correlated (Goodman & Scott, 1999).

### Long-Term Outcomes of Childhood Dysregulation Profile (DP)

Evidence from twin studies suggests that childhood DP is highly heritable (Althoff et al., 2006) and remains stable from childhood into adulthood (Aebi et al., 2020). It has been consistently associated with adverse later-life outcomes, including risk of suicidal ideation and attempts, self-harm (Caro-Cañizares et al., 2019), cannabis use (de Genna et al., 2013), ADHD and mood disorders (Masi et al., 2015) up to 14 years later (Althoff et al., 2010). Children with DP also tend to exhibit higher levels of hostility, risk taking, callousness, and impulsiveness during adolescence (de Caluwé et al., 2013). In clinical settings, many children referred to child and adolescent mental health services present with comorbid emotional and behavioural dysregulation (Wang, 2022). Together, these findings suggest that childhood DP could be an early developmental risk marker for persisting self-regulation difficulties and severe psychopathology across the lifespan (Holtmann et al., 2011b). However, the factors contributing to the development and persistence of childhood DP remain poorly understood.

### Current Study

Existing research has identified various potential predictors of childhood DP, but a comprehensive systematic understanding is lacking. A previous systematic review (Caro-Cañizares et al., 2015) found that genetic heritability, differences in brain functioning, parental substance use, and parenting behaviours were associated with childhood DP. However, the review used broad and limited search terms, potentially missing relevant studies. It included both cross-sectional and longitudinal designs but focused only on three studies specifically examining childhood DP, limiting the generalizability of its findings. Since then, numerous relevant studies have emerged. Therefore, a new systematic review is needed to provide an updated and more thorough examination of early predictors of childhood DP. The current review aims to identify key child- and family-related predictors of childhood DP within non-clinical populations, to better understand early-emerging risk factors prior to the onset of clinical symptoms.

## Methods

This systematic review was registered with the PROSPERO International Prospective Register of Systematic Reviews (registration no: CRD42024588573) and was conducted in line with the PRISMA guidelines (Moher et al., 2010).

### Study Selection Criteria

Prospective longitudinal studies were eligible for inclusion in this systematic review. Studies were included in the review based on four criteria. First, they had to report on the childhood dysregulation profile (DP) along with at least one predictor. Second, participants had to have a mean age of 18 years or younger at the time of the assessment of the dysregulation profile. Third, studies had to report on child or family-related predictors assessed during early childhood (0–8 years). Child-related predictors refer to individual characteristics of the child, whereas family-related predictors refer to aspects of the child’s wider environment influenced by familial circumstances. Fourth, the articles had to be published in a peer-reviewed journal in English.

Studies were excluded if they included a clinical sample (e.g., participants with a primary diagnosis of anxiety, autism spectrum disorders) or at-risk sample (e.g., preterm born children). The review focused on non-clinical populations to identify predictors of childhood DP as they emerge in the general population, avoiding potential confounding influences of clinical diagnoses, treatment exposures, or disorder-specific symptom profiles (Barkus et al., 2022; He et al., 2020). This approach aimed to improve the specificity of findings for early identification and prevention, addressing a gap in the literature, which has largely focused on clinical populations.

### Search Strategy

A literature search was conducted to identify longitudinal studies examining predictors of the childhood dysregulation profile published up to December 2024. The article search was finalized in January 2025. The following electronic databases were searched: PubMed, PsychINFO, MEDLINE, and Web of Science. In addition, the reference lists of included studies and the first 100 results from a grey literature search conducted via Google Scholar were screened for additional relevant studies. Grey literature refers to materials published through non-traditional channels, often without peer-review, but which may still provide valuable evidence (Mahood et al., 2014).

The keywords used were as follows: ("childhood dysregulation" OR "CBCL dysregulation" OR "SDQ dysregulation" OR "dysregulation profile" OR “CBCL juvenile bipolar” OR “CBCL-juvenile bipolar” OR “Child Behavior Checklist–Juvenile Bipolar” OR “Child Behaviour Checklist–paediatric Bipolar” [Abstract and Title]) AND (predict* OR indicat* OR “risk factor*” [Full text]).

### Quality Assessment

The Newcastle–Ottawa Scale (Wells et al., 1999) was used to assess the quality of studies referring to selection, comparability, and outcome or exposure for cohort studies (see Table 1). Scores in this scale could range from 0 to 9, with higher scores indicating higher quality. Studies were rated by EV and AB.

**Table 1:** Summary of included studies

Author (year)	Sample characteristics				Study design					
	Country	N	Mean age at childhood DP assessment(s)	% Child sex	Cohort recruitment year	Name of the cohort	Childhood DP assessment instrument	Operationalization of childhood DP^a^	Assessed predictor (s)	Study quality rating
Adynski (2023)	USA	206	18, 24, 30, 36, 60, 84 months	51.5% male; 48.5% female	2002	Durham Child Health and Development Study	CBCL	A sum score of ≥ 180; Dysregulation class trajectory	**Family related:** Social adversity*	6
Asmussen (2022)	Denmark	1099	2.5 and 5 years	54.3% male; 45.8% female	2010–12	Odense Child Cohort	CBCL	≥ 11 points, equalling the 75% percentile	**Child related:** Sex, Gestational age, Birth weight **Family related:** Maternal age,* Paternal age,* Maternal smoking during pregnancy,* Maternal depression symptoms,* Paternal depression symptoms, Maternal education.*	7
Ayer (2013)	The Netherlands	489	6–16 years	47% male; 53% female	2003–05	Not available	CBCL	80% percentile	**Child related:** Sex	8
Basten (2013)	The Netherlands	6131	5–7 years	50.3% male; 40.7% female	2002–06	Generation R	CBCL	Latent class analysis: Highly problematic class	**Child related:** Sex.* **Family related:** Maternal education level,* Psychological symptoms of both parents at 3 years,* Parental hostility.*	7
Bilgin (2024)	UK	16,599	3, 5, 7, 11 years	51.2% male; 48.8% female	2000–02	Millennium Cohort Study (MCS)	SDQ	Parallel-process latent class analysis: Co-developing low increasing internalizing and high stable externalizing trajectory class	**Child related:** Sex,* Birth weight*, Temperament at 9 months (positive mood,* withdrawal,* low adaptability, regularity) Night waking frequency at 9 months **Family related:** Maternal age at birth, Structured parenting beliefs, Maternal psychological distress at 9 months*	8
Deutz (2020)	USA	1073	8 and 14 years	50.2% male; 49.8% female	1991	NICHD Study of Early Child Care and Youth Development (SECCYD)	CBCL	Confirmatory Factor Analysis	**Child related:** Birth weight, Attachment, Temperament (negative affectivity*), Executive functioning (effortful control,* cognitive ability, delay of gratification*, impulsivity, planning/problem solving, self-control.*) **Family related:** Positive maternal parenting,* harsh control, Maternal depression,* Home environment	6
Frazier (2023)	USA and Puerto Rico	4595	6–8 years 9–11 years 12–14 years 15–18 years	47.1% female 52.9% male	2009–2021	Environmental influences on Child Health Outcomes (ECHO)	CBCL	Sum score ≥ 180	**Child related:** Sex,* Small for gestational age **Family related:** Prenatal tobacco use,* Physical health (Any prenatal infection,* Gestational diabetes), Maternal education level,* Family psychiatry history.*	7
Herbein (2024)	France	871	3, 5 and 8 years	47% female 53% male	2003–06	EDEN cohort	SDQ	Latent class growth analysis: High mood dysregulation trajectory	**Child related:** Sex,* Gestational age, Birth weight **Family related**: Maternal and paternal age, Maternal smoking and alcohol use during pregnancy, Depression symptoms at 6 months during pregnancy.*	7
Hofheimer (2023)^b^	USA and Puerto Rico	3934	Across 4 time points from 18 to 72 months	53.2% male; 46.8% female	2009–2021	Environmental influences on Child Health Outcomes (ECHO)	CBCL	Sum score ≥ 180; High and increasing DP trajectory	**Child related:** Sex,* Gestational age.* **Family related:** Maternal age, Maternal psychiatric diagnoses,* Psychosocial adversity,* Maternal education,* Overall substance use during pregnancy (specific effects of alcohol,* nicotine* and opiod*)	7
Marino (2019)	Italy	104	2 years	49% female 51% male	Not reported	Not available	CBCL	Sum score of DP	**Child related:** Gestational age.* **Family related:** Parental age, Maternal depression symptoms.*	7
McQuillan (2018)	USA	585	Annually from 5 to 13 years	48% female; 52% male	1987–88	Child Development Project	CBCL by mothers; TRF by teachers	T score of ≥ 60	**Child related:** Temperament (resistance to control*), Social preference in peer relationships,* Language ability.* **Family related:** Harsh parenting, Stressful life events	7
Winsper and Wolke (2014)	UK	10,630	4, 7, 8 & 9.5 years	48.4% female;51.6% male	1991–92	Avon Longitudinal Study of Parents and Children (ALSPAC)	SDQ	Latent class growth analysis: Very high dysregulation trajectory class	**Child related:** Infant and toddler regulatory problems (excessive crying, sleeping and feeding problems).*	8

*DP* Dysregulation Profile; *CBCL* Child Behavior Checklist; *YSR* Youth Self Report; *SDQ* Strengths and Difficulties Questionnaire; *TRF* Teacher’s Report Form

^a^Dysregulation profile measured with the combination of the following subscales: CBCL: Emotional (anxious/depressed), cognitive (attention problems), and behavioral (aggressive behavior) subscales; YSR: Anxious/depressed, the attention problems, and the aggression subscales; SDQ: Emotional symptoms, hyperactivity/inattention, conduct problems subscales

^b^Same cohort with Frazier (2023). Included due to the report of different time points

^*^Indicates significant findings

### Data Extraction

Data were extracted using an Excel sheet and included information on author names, publication year, country of the data collection, number of participants, percentages (%) of male and female participants, age of assessment of the dysregulation profile, assessment scale of the dysregulation profile, name of the study cohort (if available), T1 (Time 1) assessment year of the cohort, and assessed predictors (child related and/or parent related).

### Evidence Synthesis

Given that quantitative synthesis was not feasible due to the lack of enough number of studies and heterogeneity across studies in design, all studies were narratively synthesised. To address the aims of our review, a narrative overview of the main findings is presented in two major sections: (1) Child-related predictors (child sex; birth weight, gestational age, and small for gestational age; child temperament; language and cognitive skills; early social development); and (2) family-related predictors (mental health; social adversity; education; parental behaviours; life stressors; age; substance use; physical health during pregnancy).

## Results

### Included Articles

**Fig. 1:**
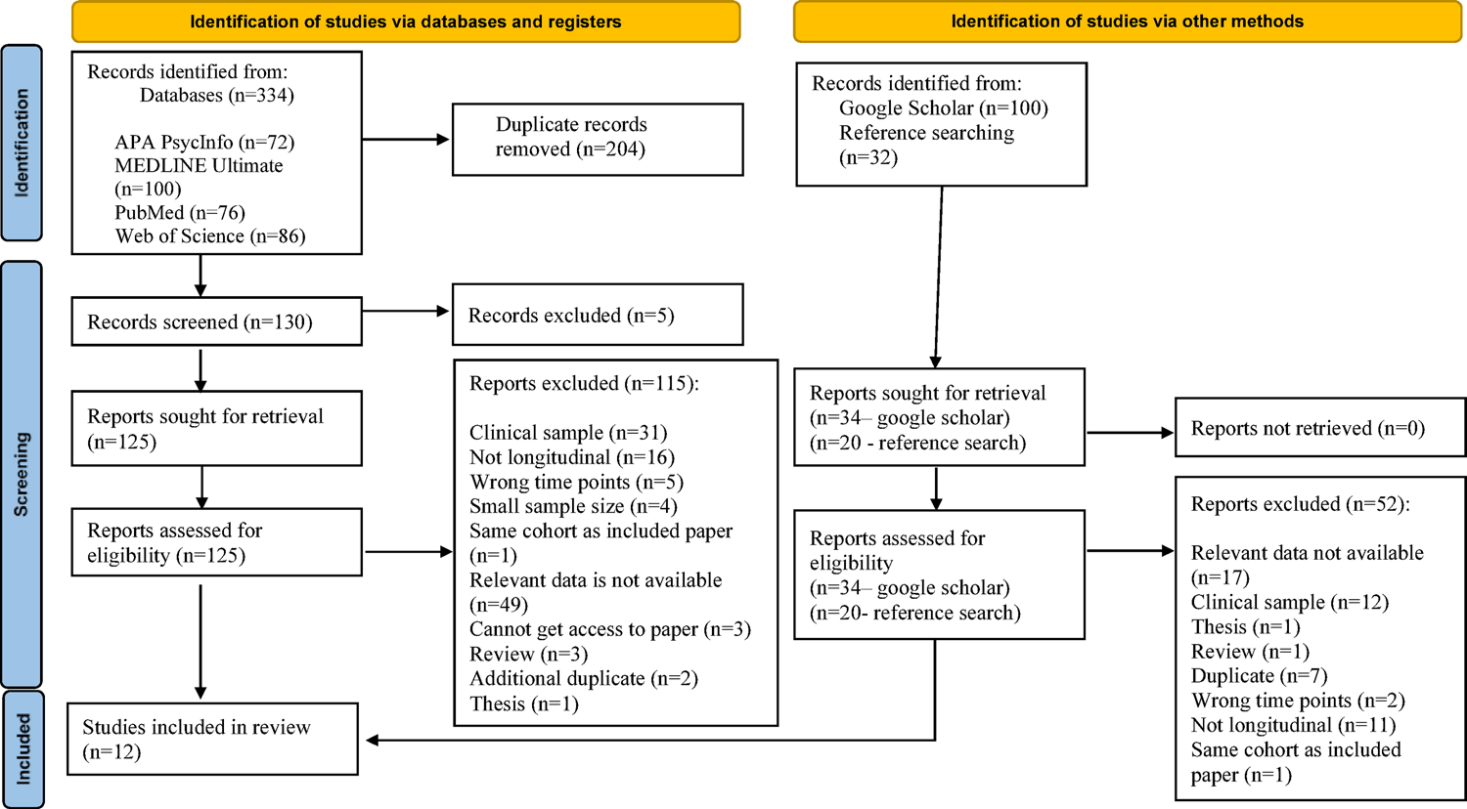
Search strategy

The PubMed search yielded 76 articles, PsychINFO yielded 72 articles, MEDLINE yielded 100 articles, and Web of Science yielded 86 articles. Furthermore, 54 articles were found from bibliography search. Overall, 388 articles were included in the literature search. After the removal of 209 duplicates, the literature search included 179 articles (see Fig. 1).

After title screening, 125 articles remained for abstract review. Based on the abstracts, 64 articles were excluded. The full texts of the remaining 61 articles were then assessed against the inclusion criteria, resulting in the exclusion of 44 studies due to one or more of the following reasons: inclusion of a clinical sample (N = 10), unsuitable study design (N = 13), insufficient or unavailable data (N = 15), participants outside the target age range (N = 1), inability to access the full text (N = 3), or small sample size (N = 2). Reference lists of included studies were reviewed, and a grey literature search of the first 100 Google Scholar results was conducted. In cases where multiple reports were published from the same cohort, only one study was included to avoid double-counting participants. When selecting between such studies, priority was given to those with the most comprehensive profiles such as largest sample sizes and a broader number of predictors. However, two studies (Frazier et al., 2023; Hofheimer et al., 2023) from the Environmental influences on Child Health Outcomes (ECHO) cohort were included as they reported on different time points. This resulted in 12 articles from 10 samples being included in the review (Table 1). The article selection process was conducted independently by EV and AB, with discrepancies at the abstract and full-text screening stages discussed and resolved collaboratively.

### Study Characteristics and Quality Assessment

Majority of the studies (N = 9, 75%) reported on both child- and family-related predictors of childhood dysregulation, while 2 (17%) studies reported on child-related predictors only, and 1 reported on family-related predictors only. The number of participants ranged from 104 to 16,599. Majority of the studies were conducted in Europe (N = 7, 58%), and 5 (42%) were conducted in the USA and Puerto Rico. The assessment ages of childhood dysregulation ranged from 18 months to 18 years. Majority of the studies used CBCL to assess childhood dysregulation (N = 9, 75%) and 3 (25%) studies used SDQ. The quality assessment indicated low risk of bias in sample selection, low-moderate risk of comparability bias, and low risk of exposure/outcome bias. Overall quality ratings of the studies ranged from 6 to 8 (mean = 7.1, SD = 0.67), indicating overall high quality.

### Predictors of Childhood Dysregulation Profile

**Fig. 2:**
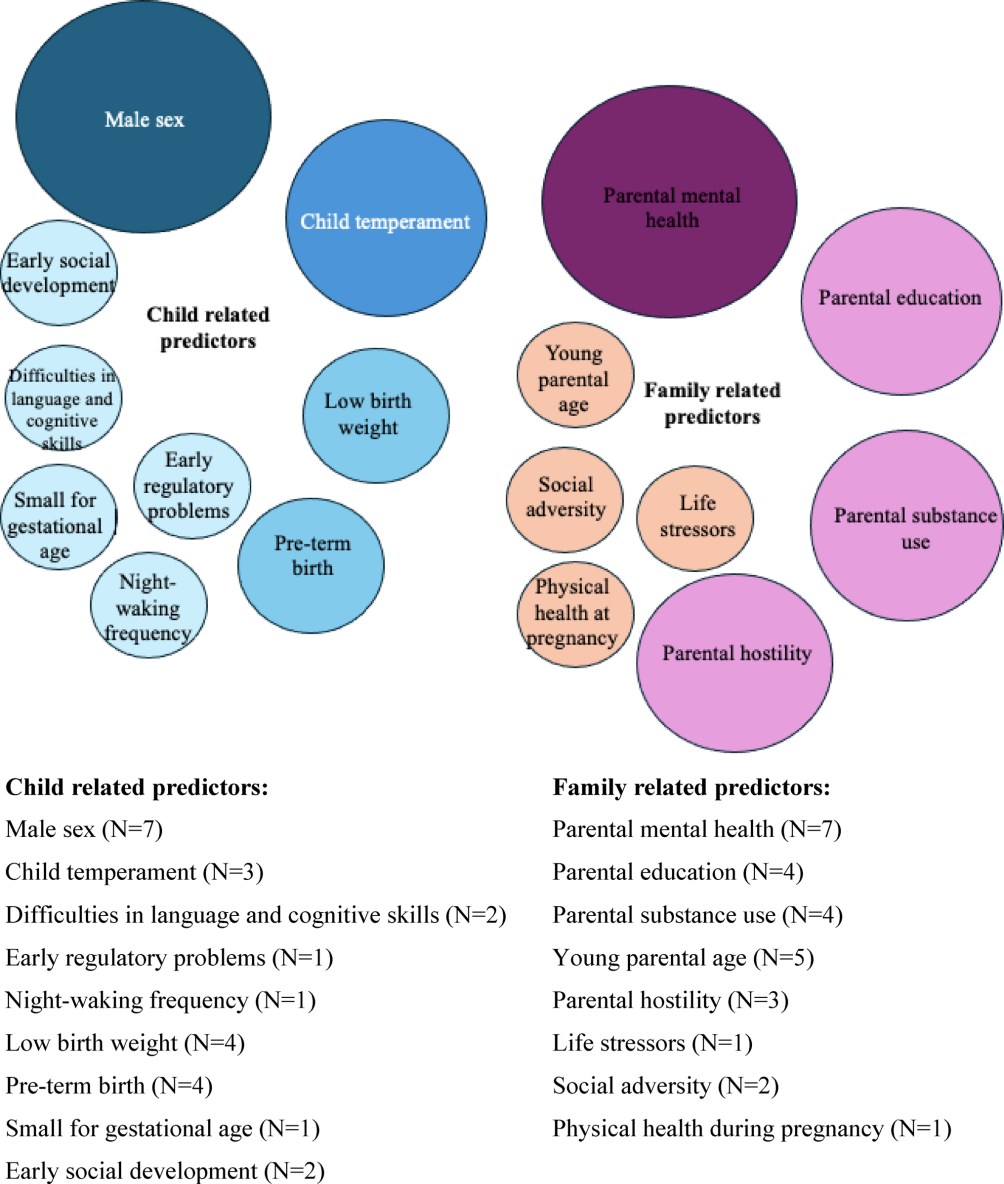
Key child related (blue) and family related (pink) predictors of childhood DP

Figure 2 illustrates the key child-related (blue) and family-related (pink) predictors of childhood DP. Larger circles with darker colours represent predictors supported by a greater number of studies and more consistent findings. In contrast, smaller circles with lighter colours indicate predictors with fewer studies or inconsistent evidence regarding their association with childhood DP.

### Child Related Predictors

Studies assessed several child-related predictors of childhood DP with most focusing on infant sex, birth weight, and gestational age. Other predictors included temperament, language ability, social preference, executive functioning skills, infant-parent attachment, and infant/toddler regulatory problems.

#### Child Sex

Seven studies (Asmussen et al., 2022; Ayer et al., 2013; Basten et al., 2013; Bilgin et al., 2024; Frazier et al., 2023; Herbein et al., 2024; Hofheimer et al., 2023) examined child sex as a predictor of childhood DP. Four studies (Basten et al., 2013; Frazier et al., 2023; Herbein et al., 2024; Hofheimer et al., 2023) found that boys are more likely than girls to show childhood DP symptoms. For example, Hofheimer et al. (2023) reported that boys are more likely to have higher and increasing DP symptoms trajectories from 18 to 72 months (in USA), and Herbein et al. (2024) showed that they are more likely to be on a high mood dysregulation trajectory from 3 to 8 years of age (in France). Similarly, Basten et al. (2013) found more DP symptoms in boys aged 5–7 years (in the Netherlands). Across 3 to 11 years, Bilgin et al. (2024) showed that boys were more likely to be in the high DP (co-developing low increasing internalizing and high stable externalizing) trajectory class in comparison to girls (in the UK). Using data from 69 paediatric longitudinal cohorts in the USA and Puerto Rico, Frazier et al. (2023) showed that boys are more likely to show childhood DP symptoms at ages 6 to 18 years in comparison to girls.

Conversely, two studies found no significant sex differences regarding childhood DP symptoms. Asmussen et al. (2022) reported no differences in high-persisting DP trajectories between boys and girls from 2.5 to 5 years (in Denmark). Ayer et al. (2013) also found no sex differences in DP symptoms from 11.5 to 14.5 (the Netherlands), with boys comprising 47% and girls 43% of the persistent DP group.

#### Child Temperament

Three studies (Bilgin et al., 2024; Deutz et al., 2020; McQuillan et al., 2018) consistently found that child temperament influences the development of childhood DP symptoms. Bilgin et al. (2024) reported that infant temperament at 9 months predicted a high DP symptom trajectory from ages 3 to 11, with positive mood and regularity reducing, and withdrawal increasing, the likelihood of high DP symptoms. Deutz et al. (2020) found that negative affectivity was associated with higher DP symptoms at ages 8 and 14. McQuillan et al. (2018) showed that resistance to control predicted higher DP symptoms in both mother (*r* = 0.15) and teacher reports (*r* = 0.05).

#### Birth Weight, Gestational Age, and Small for Gestational Age

Four studies (Asmussen et al., 2022; Bilgin et al., 2024; Deutz et al., 2020; Herbein et al., 2024) examined birth weight, four (Asmussen et al., 2022; Herbein et al., 2024; Hofheimer et al., 2023; Marino et al., 2019) examined gestational age, and one study (Frazier et al., 2023) assessed small for gestational age as predictors of childhood DP.

Regarding birth weight, Bilgin et al. (2024), using a population representative cohort in the UK, found that higher birth weight was associated with a lower likelihood of being in a high DP symptom trajectory from ages 3 to 11. In contrast, three smaller cohort studies found no significant associations between birth weight and childhood DP symptoms. These included Asmussen et al., (2022; N = 1099), which found no impact of birth weight on the likelihood of high persistent DP symptoms from 2.5 to 5 years. Similarly, Herbein et al., (2024; N = 871) and Deutz et al., (2020; N = 1073) reported no impact of birth weight on DP symptoms from 3 to 8 years and at ages 8 and 14 years, respectively.

Findings on gestational age were mixed. Marino et al. (2019) reported increased DP symptoms with decreasing gestational age at 2 years. Hofheimer et al. (2023) found that preterm children were more likely to follow a high and increasing DP trajectory than those born full-term. Conversely, Herbein et al. (2024) and Asmussen et al. (2022) found no associations between gestational age and childhood DP symptoms.

Regarding small for gestational age, Frazier et al. (2023) reported no associations with DP symptoms between ages 6 to 18 years.

#### Language and Cognitive Skills

One study (McQuillan et al., 2018) explored the association between child language ability and change in childhood DP symptoms from ages 5 to 13. Results showed that children with more advanced language skills exhibited less increase in DP symptoms based on teacher or combined teacher and mother reports. However, no association was found when DP was reported by mothers alone.

Regarding cognitive skills, Deutz et al. (2020) showed that lower effortful control, cognitive ability and self-control were associated with higher DP symptoms at ages 8 and 14, with effortful control showing relatively larger associations (*r* = −0.28 at 8 years and *r* = −0. 21 at 14 years).

#### Early Social Development

Regarding infant-mother attachment, Deutz et al. (2020) found no associations between insecure or disorganised attachment from birth to 54 months, and DP symptoms at ages 8 and 14. In contrast, McQuillan et al. (2018) reported that children with low social preference in early peer relationships (between grades 1 to 3) showed steeper growth in DP symptoms from ages 5 to 13.

#### Early Regulatory Problems (Excessive Crying, Sleeping, and Feeding Problems) and Night-Waking Frequency

Using a large-scale, prospective UK population representative data (recruitment years 1991–92), Winsper and Wolke (2014) found that regulatory problems at 6, 15–18 and 24–30 months were consistently associated with higher likelihoods of childhood DP symptom trajectories from ages 4 to 9.5. The strength of the associations increased as the age at the assessment of the regulatory problems increased (e.g., strongest associations found for 24–30 months regulatory problems), as the severity of the DP symptom trajectories increased (i.e., low, moderate, high, very high DP), and as the severity of the regulatory problems increased (i.e., single, multiple regulatory problems). In contrast, using population representative large-scale data from a later UK cohort study (recruitment years 2000–02), Bilgin et al (2024) showed no association between night waking frequency at 9 months of age and childhood DP symptom trajectories from 3 to 11 years.

### Family Related Predictors

#### Parental Mental Health

Eight studies investigated the association between parental mental health and childhood DP symptoms. Among these, four studies (Asmussen et al., 2022; Deutz et al., 2020; Herbein et al., 2024; Marino et al., 2019) focused on maternal depression, while three studies (Basten et al., 2013; Frazier et al., 2023; Hofheimer et al., 2023) examined broader indicators of family mental health and one study examined maternal distress (Bilgin et al., 2024) in relation to childhood DP.

Across all studies, a consistent pattern emerged linking poorer parental mental health to higher levels of childhood DP symptoms. Regarding maternal depression, Deutz et al. (2020) found a significant association between postnatal maternal depression and elevated childhood DP symptoms at both 8 and 14 years (*p* = 0.03 for both). Similarly, Herbein et al., 2024 reported that maternal clinical depression during pregnancy was associated with a 44% likelihood of children following a persistent mood dysregulation trajectory (44%) between ages 3 and 8. Bilgin et al. (2024) also identified a significant association between maternal psychological distress at 9 months postpartum and child dysregulation from ages 3 to 11. Marino et al. (2019) provided a neurobiological explanation for these findings, demonstrating that children of mothers with high levels of postnatal depression exhibited left parietal alpha asymmetry, which is an indicator linked to greater levels of emotional dysregulation.

Asmussen et al., (2022) examined both maternal and paternal depression symptoms. They found that higher maternal depressive symptoms at 3 months postpartum predicted persistent childhood DP symptoms from 2.5 to 5 years, whereas paternal depressive symptoms at the same time point were not significantly associated with childhood DP trajectories.

Studies examining broader parental mental health difficulties revealed similar patterns. In a large sample of 6,131 children, Basten et al. (2013) found higher levels of both maternal and paternal postnatal affective symptoms (i.e., depression and anxiety) to be positively correlated with childhood DP. Additionally, Hofheimer et al. (2023) reported that current or historical maternal mental health difficulties, including major depression, dysthymia, phobias, and bipolar, anxiety, panic, obsessive–compulsive, post-traumatic stress, and attention-deficit disorders, were associated with both high and increasing (50%) and borderline but stable (29.4%) childhood DP trajectories. Frazier et al. (2023) further demonstrated that children with DP were more likely to have at least one parent with a psychiatric disorder (major depression, dysthymia, bipolar disorder, anxiety disorder not otherwise specified, generalized, anxiety disorder, specific phobia, panic disorder, obsessive–compulsive disorder, social anxiety, post-traumatic stress disorder, attention-deficit/hyperactivity disorder, eating disorder, schizophrenia, alcoholism or other substance abuse, and autism spectrum disorder) compared to children without DP (68% vs. 50%). However, the timing of parental psychiatric diagnoses was not clearly specified in this study.

#### Parental Education

Four studies (Asmussen et al., 2022; Basten et al., 2013; Frazier et al., 2023; Hofheimer et al., 2023) investigated the association between parental education and childhood DP symptoms, revealing consistent findings across samples. In a longitudinal study following children from age 6 to 18 years in the United States and Puerto Rico, Frazier et al. (2023) found that children whose mother had less than a high school education were more likely to exhibit higher DP trajectories compared to those whose mothers had completed a university degree. Similarly, Basten et al. (2013) reported that children aged 5–7 years whose mothers had lower educational attainment (i.e., primary school or lower vocational education) displayed higher levels of childhood DP. Consistent with these results, Asmussen et al. (2022), and Hofheimer et al. (2023) also found that children of mothers with less than a college-level education were more likely to exhibit high or borderline DP levels.

#### Maternal Substance Use During Pregnancy

Four studies (Asmussen et al., 2022; Frazier et al., 2023; Herbein et al., 2024; Hofheimer et al., 2023) investigated the association between maternal substance use during pregnancy and childhood DP symptoms. Regarding maternal smoking during pregnancy, Asmussen et al. (2022) found an increased likelihood of children following a high-persisting or increasing DP trajectory. In addition, in a sample of 4,595 children aged 6 to 18 years, Frazier et al. (2023) reported that prenatal maternal tobacco exposure was associated with higher levels of childhood DP. In contrast, Hofheimer et al. (2023), in a sample of 3,934 infants aged 18–72 months, found no significant associations between overall substance use during pregnancy and childhood DP. However, specific effects were observed for alcohol (*p* <  0.001), nicotine (*p* < 0.01) and opioid use (*p* < 0.04), each of which increased the likelihood of a high and increasing DP symptom trajectory. On the other hand, Herbein et al. (2024) did not find significant associations between maternal alcohol consumption and/or cigarette smoking during pregnancy with childhood DP symptoms.

#### Parental Age

Five studies (Asmussen et al., 2022; Bilgin et al., 2024; Herbein et al., 2024; Hofheimer et al., 2023; Marino et al., 2019) examined parental age as a predictor of childhood DP. Asmussen et al. (2022) found that young motherhood (17–27 years) and young fatherhood (18–28 years) were associated with a high-persisting and increasing trajectory of childhood DP*.* In contrast, the remaining studies did not identify a significant association between maternal age at birth (Bilgin et al., 2024; Herbein et al., 2024; Hofheimer et al., 2023), paternal age at birth (Herbein et al., 2024), or overall parental age (Marino et al., 2019) and childhood DP symptoms.

#### Parenting and Family Environment

Three studies (Basten et al., 2013; Deutz et al., 2020; McQuillan et al., 2018) investigated the association between parenting and childhood DP symptoms. Basten et al. (2013) found a significant association between higher levels of parental hostility and elevated childhood DP symptoms (*p* <  0.001 for both mothers and fathers). Similarly, Deutz et al. (2020) reported a negative association between positive maternal parenting and childhood DP (*p* <  0.001). However, both Deutz et al. (2020) and McQuillan et al. (2018) found no significant association between harsh parental control and childhood DP.

Beyond specific parenting behaviours, the broader home environment also showed mixed associations. Deutz et al. (2020) also found that a poorer quality home environment was associated with higher levels of childhood DP. In contrast, McQuillan et al. (2018) did not identify a significant relationship between stressful life events and childhood DP.

In addition to parenting behaviours, Bilgin et al. (2024) examined parenting beliefs and found no significant association between structured parenting beliefs at 9 months and childhood dysregulation trajectories from ages 3 to 11 years.

#### Social Adversity

Two studies (Adynski et al., 2023; Hofheimer et al., 2023) explored social adversity as a predictor of childhood DP. Hofheimer et al., (2023) found that the majority of children experiencing multiple social adversities (between 2–5) also exhibited high and increasing (86.2%) and borderline and stable (71.3%) DP trajectories *(p* < .001)*.* Similarly, in a sample of infants aged 18–84 months, Adynski et al. (2023) reported that higher levels of social adversity were positively associated with elevated childhood DP symptoms (*p* < .001), although they were not significantly associated with DP trajectories.

#### Physical Health During Pregnancy

One study (Frazier et al., 2023) examined the impact of physical health during pregnancy on development of childhood DP. The authors found prenatal infections to be associated with childhood DP.

## Discussion

The aim of this systematic review was to investigate the evidence for predictors of childhood DP, focusing on family- and child-related factors separately. Several family- and child-related predictors emerged including poorer parental mental health, lower parental education, greater substance use during pregnancy, social adversity, male sex, difficult temperament, and early regulatory problems. These findings provide an overview of factors associated with the development of childhood DP.

Consistent with previous developmental psychopathology research (Babineau et al., 2015), poor parental mental health emerged as a key predictor of childhood DP symptoms, underscoring the critical role parents play in modelling and supporting the development of self-regulation skills (Morawska et al., 2019). Parental mental health difficulties, particularly in the postnatal period, could decrease parents’ capacity to regulate their own emotions, thereby limiting their ability to help earn how to self-regulate their emotions and behaviours (Zitzmann et al., 2024). The risk for childhood DP could depend not only on the presence of parental mental health symptoms but also on the timing of parental mental health symptoms (Papp, 2012). Existing studies mainly investigated the impact of mental health symptoms of mothers in the postnatal period on the development and maintenance of childhood DP symptoms. Further research is required to examine the impact of mental health symptoms both in the prenatal and postnatal period including both mothers and fathers in the study.

Relatedly, evidence on the impact of parenting behaviours on the development of childhood DP symptoms remains mixed (Basten et al., 2013; Deutz et al., 2020; McQuillan et al., 2018), possibly due to variations in the developmental stages examined as the impact of negative parenting on the development of childhood DP could be more observable in the early years when childhood DP is emerging. It could also be due to the bidirectional association between parenting behaviours and childhood DP symptoms which is often overlooked.

Although research directly examining the association between parenting and childhood DP symptoms is limited, evidence from studies on the related construct of emotion dysregulation provides a valuable context. A review of decades of research on childhood emotion dysregulation indicates that self-regulation skills are transmitted from parents to children (Bridgett et al., 2015), with meta-analyses showing small but significant associations between negative parenting and emotion dysregulation in childhood (Zimmer-Gembeck et al., 2022) and in adolescence (Goagoses et al., 2023). Stronger associations have been found between parents’ emotion regulation difficulties (e.g., low emotional awareness, suppression, limited strategies) and children’s internalizing problems, while associations between parents’ adaptive regulation skills (e.g., cognitive reappraisal) and children’s externalizing behaviours appear to be weaker (Zimmer-Gembeck et al., 2022). Collectively, these findings indicate that parental mental health and emotion regulation capacities play a central role in shaping children’s emotion regulation development and underscore the need for further research on their contribution to childhood DP.

The current review provides new evidence that had not been identified in the previous review by Caro-Cañizares et al., 2015. It highlights that low maternal education is associated with a greater likelihood of both the development and persistence of childhood DP symptoms (Asmussen et al., 2022; Basten et al., 2013; Frazier et al., 2023; Hofheimer et al., 2023). This association could reflect broader contextual factors such as lower socioeconomic status, reduced social support network and limited knowledge of effective parenting techniques (Kondirolli & Sunder, 2022; Song, 2023). Furthermore, maternal smoking during pregnancy emerged as a key factor predicting the likelihood and trajectory of childhood DP, aligning with previous research linking maternal prenatal smoking to a range of adverse developmental outcomes in children (Chen, 2012; Knopik et al., 2012). This review also highlighted the impact of cumulative risk exposure (e.g., number of children in the household, low maternal education, single parenting, presence of maternal mental health problems), collectively reflecting a greater level of social adversity. Consistent with prior findings, higher levels of social adversity are associated with an increased likelihood of childhood DP (Adynski et al., 2023; Hofheimer et al., 2023).

Regarding child-related predictors of childhood DP, male sex, difficult temperament (e.g., negative mood, low regularity, resistance to control), and early regulatory problems. (i.e., excessive crying, sleeping, or feeding problems) emerged as key factors associated with a higher likelihood of developing and maintaining DP symptoms. The association between these factors and the development of childhood DP symptoms may be explained by their potential impact on the formation of secure attachment with parents (Bilgin & Wolke, 2020), which in turn has been linked to the emergence of DP symptoms (Groh et al., 2012). Furthermore, difficult temperament and early regulatory problems may negatively influence sensitive parenting (Jaekel et al., 2021), which could mediate the association between these factors and later DP symptoms. Another plausible explanation involves underlying neurobiological mechanisms (e.g., differences in amygdala activity) that might contribute to these associations (Álvarez-Voces & Romero, 2025; Sammallahti et al., 2023; Urben et al., 2024).

On the other hand, the evidence regarding the effects of low birth weight, and gestational age on the development of childhood DP remains inconclusive. This inconsistency might reflect the differential of preterm birth and low birth weight on internalizing rather than externalizing symptoms (Bilgin et al., 2021). However, further research is required to clarify these mixed findings. In addition, the limited evidence concerning the roles of early language ability, cognitive skills and social development makes it difficult to draw conclusions about their potential contribution to the development of DP symptoms, warranting further investigation.

### Comparison with Other Reviews

A previous systematic review (Caro-Cañizares et al., 2015) examined predictors of childhood DP but used broad search terms and included only a few studies specifically on this topic, limiting the generalizability of its findings. Consistent with that review, the current review identified parental behaviours, substance use, and psychological well-being as robust predictors, with multiple studies supporting each factor. Caro-Cañizares et al. (2015) also reported biological predictors (e.g., genetics, blunted HPA-axis responses); however, these findings were derived from cross-sectional data and were not included in the current review. In the current review, two longitudinal studies examined biological predictors of childhood DP. Firstly, Herbein et al. (2024) found lower levels of tumour necrosis factor-α at birth to be associated with higher levels of childhood mood dysregulation. Additionally, Marino et al. (2019) identified that mothers who experienced postnatal depression were more likely to have children with greater left parietal alpha asymmetry at 6 months of age, which later predicted higher levels of emotional dysregulation at 2 years of age. However, due to the limited number of longitudinal studies examining biological predictors, these biological factors could not be integrated into the current systematic review. Given the evidence that neural activity, thyroid function, and genetic risk influence childhood DP (Althoff et al., 2006; McGough et al., 2013; Tsai et al., 2024; Zepf et al., 2011), our findings highlight the need for further longitudinal studies investigating biological predictors of childhood DP. Differences between the reviews also reflect the focus on the study design, as Caro-Cañizares et al. (2015) mainly included cross-sectional studies. One longitudinal study from that review (Dougherty et al., 2014) was excluded here due to unavailable data on predictor factors.

### Clinical Implications

The findings of the current study have important clinical implications, as they identify a range of modifiable family-related predictors of childhood DP. These findings highlight the importance of implementing family-based interventions to support children and families affected by DP. For example, multi-component interventions that focus on enhancing both child and parental emotional sensitivity, as well as addressing broader family difficulties such as the Turning into Kids and the Triple P-Positive Parenting Program (Havighurst et al., 2013; Sanders, 2012) could be particularly beneficial for children exhibiting DP symptoms.

### Limitations

To our knowledge, this is the first comprehensive review to examine predictors of childhood DP, highlighting the key child- and family-related factors. However, several limitations should be acknowledged. First, there was considerable heterogeneity in measurement of DP (e.g., latent class profiling, 75th percentile, 80th percentile), and timing of assessments, making it difficult to compare the study findings. Second, most studies did not account for potential bidirectional processes between child behaviour and family factors. Third, the review was limited to studies published in English-language peer-reviewed journals, which may have resulted in exclusion of studies published in other languages. Further studies are needed in non-Western contexts to determine whether these findings generalize across diverse cultural settings.

## Conclusions

To conclude, this review identified several predictors of childhood DP. Family-related predictors, including poorer parental mental health, lower parental education and greater maternal substance use during pregnancy emerged as key risk factors. In addition, child characteristics such as male sex, difficult temperament and regulatory problems were associated with an increased likelihood of developing DP symptoms. Importantly, many of the family-related factors are modifiable, underscoring the potential value of family-based interventions to support children and families affected by DP. Understanding modifiable early-life predictors is essential for identifying children at elevated risk and informing early intervention efforts. Future research should aim to replicate these findings in non-Western populations and explore additional contextual and cultural influences on the development of childhood DP.

## Data Availability

No datasets were generated or analysed during the current study.
